# Natural-Based Antioxidant Extracts as Potential Mitigators of Fruit Browning

**DOI:** 10.3390/antiox9080715

**Published:** 2020-08-07

**Authors:** Cindy Dias, Alexandre M. A. Fonseca, Ana L. Amaro, Ana A. Vilas-Boas, Ana Oliveira, Sonia A. O. Santos, Armando J. D. Silvestre, Sílvia M. Rocha, Nélson Isidoro, Manuela Pintado

**Affiliations:** 1 Centro de Biotecnologia e Química Fina—Laboratório Associado, Escola Superior de Biotecnologia, Universidade Católica Portuguesa/Porto, Rua de Diogo Botelho, 1327, 4169-005 Porto, Portugal; cdias@porto.ucp.pt (C.D.); aamaro@porto.ucp.pt (A.L.A.); avboas@porto.ucp.pt (A.A.V.-B.); asoliveira@porto.ucp.pt (A.O.); 2 CICECO-Instituto de Materiais de Aveiro, Departamento de Química, Universidade de Aveiro, 3810-193 Aveiro, Portuga; alexandrefonseca@ua.pt (A.M.A.F.); santos.sonia@ua.pt (S.A.O.S.); armsil@ua.pt (A.J.D.S.); 3 LAQV-REQUIMTE, Departamento de Química, Universidade de Aveiro, 3810-193 Aveiro, Portugal; smrocha@ua.pt; 4 Cooperativa Agrícola dos Fruticultores do Cadaval, CRL (COOPVAL), Estrada Nacional 115, Km 26 2550-108 Cadaval, Portugal; nelson.isidoro@coopval.com

**Keywords:** natural-based extracts, enzymatic browning, phenolic compounds, antioxidant activity

## Abstract

Fruit enzymatic browning (EB) inhibition continues to be a challenge in the Food Industry. This physiological disorder results mainly from the oxidation of natural phenolic compounds by polyphenoloxidase (PPO) and peroxidase (POX) leading to the formation of brown pigments. EB can be controlled with the application of antioxidants, reducing/inhibiting the activity of these oxidative enzymes. In this study, strawberry tree (leaves and branches) and apple byproduct were the natural-based extracts (NES) selected, as potential tissue browning inhibitors, within a first screening of fifteen natural-based extracts with antioxidant properties. Phenolic profile, total phenolic content and antioxidant activity of the selected extracts were also performed as well as their depletion effect on the oxidative enzyme’s activity and browning inhibiton in fresh-cut pears. Strawberry tree extracts (leaves and branches) revealed higher total phenolic content (207.97 ± 0.01 mg GAE.g_NES_^−1^ and 104.07 ± 16.38 mg GAE.g_NES_^−1^, respectively), confirmed by the plethora of phenolic compounds identified by LC-ESI-UHR-QqTOF-HRMS and quantified by HPLC. This phytochemical composition was reflected in the low IC_50_ against PPO and POX obtained. Despite the lower phenolic content (6.76 ± 0.11 mg GAE.g_NES_^−1^) and antioxidant activity (IC_50_ = 45.59 ± 1.34 mg mL^−1^), apple byproduct extract showed potential in delaying browning. This study highlights the opportunity of byproducts and agricultural wastes extracts as novel anti-browning agents.

## 1. Introduction

In order to maintain the competitiveness of horticultural products, the preservation of their quality during postharvest processing and storage is of utmost importance, since it extends shelf-life and therefore the marketing period to keep a high selling price [1]. However, during this phase, several physiological disorders, responsible for the deterioration of fresh produce organoleptic properties might occur. 

Enzymatic browning (EB) is one of such disorders and is considered a serious problem for the Food Industry as it represents the second most important cause of fresh produce deterioration [2]. EB occurrence has a negative impact on color, taste, flavor, and nutritional value, leading to faster quality deterioration and shorter shelf-life [2,3]. EB development has been associated with polyphenoloxidase (PPO) and peroxidase (POX) activities. PPO catalyze the dehydrogenation of *o*-dihydroxy phenols to *o*-quinones [4,5]. These quinones react with each other and surrounding proteins leading to the formation of melanin, responsible for the dark color that characterizes EB in fruits [6]. POX catalyze H_2_O_2_ oxidation of OH groups of phenolic compounds present in fruits [6].

However, none of these reactions occurs in intact plant cells, since substrates are located in vacuoles while enzymes are in the cytoplasm without contact. Therefore, EB only takes place once cell compartmentalization is compromised and enzymes become in contact with substrates in the presence of oxygen. Besides cutting and mechanical damage, the loss of membrane integrity is associated with several stress factors that ultimately lead to an increase in reactive oxygen species (ROS) formation [7]. 

To prevent and/or attenuate this phenomenon, various procedures have been developed, namely the use of antioxidant solutions aiming at either inactivate PPO and POX or to avoid contact between the enzyme and its substrate. Antioxidant solutions, such as ascorbic acid and its derivatives and sulfites, have been traditionally employed since they were found to be the most effective in controlling browning [8]. However, regardless of their efficacy, there is an increasing consumer demand for synthetic compounds’ replacement by natural and more sustainable compounds as food ingredients [9,10,11]. The use of compounds of natural origin with anti-browning capacity would be of indubitable importance for more widespread consumers’ acceptance of these products.

Thus, the interest in natural antioxidants has increased in recent years, especially in plant phenolic compounds [12]. Phenolic compounds display strong antioxidant activity and therefore potential as oxidative enzyme inhibitors. The antioxidant activity of phenolic compounds is mainly due to their redox properties, which allow them to act as reducing agents, hydrogen donators, and singlet oxygen quenchers [13].

Another advantage of using plant phenolic compounds is their availability in plant byproducts that are often undervalued in AgroFood Industries. This potential was demonstrated by Vijayalaxmi et al. [14] using several agricultural byproducts such as those resulting from sugarcane bagasse, corn husk, peanut husk, rice bran, and wheat bran production as a cost-effective source of antioxidants. Babbar et al. [15] also reviewed the antioxidant potential and phenolic composition of fruit byproducts as well as their application as nutraceuticals resources and bio-preservatives.

In this perspective, the current study was designed to select the most promising natural-based extracts from a range of fifteen different extracts obtained from natural sources. Firstly, a screening of fifteen different plant extracts with potential in inhibiting tissue browning was carried out using sliced “Rocha” pear (*Pyrus communis* L. cv. Rocha) as a model to evaluate browning development. The obtained results allowed the selection of three novel natural-based extracts (NES), notably strawberry tree (*Arbutus Unedo* L.) (from leaves and branches), and apple pomace byproduct. NES were further investigated regarding their total phenolic content and antioxidant activity, as well as their phenolic composition in order to identify the presumed compounds responsible for their anti-browning capacity. To the best of our knowledge, this is the first time the potential of these three extracts in browning mitigation has been presented. 

## 2. Materials and Methods

### 2.1. Chemicals

HPLC-grade methanol (≥99.9% purity) was supplied by Honeywell Riedel-de Haën AG, Seelze, Germany. The commercial enzymes, peroxidase from horseradish and tyrosinase from mushroom, ABTS (2,20- azinobis (3-ethylbenzothiazoline-6-sulfonic acid) diammonium salt), 1,2-dihydroxybenzene (Catechol), guaiacol, sodium carbonate, arbutin, rutin, gallic, caffeic, ellagic, syringic and trifluoroacetic (TFA) acids were purchased from Sigma-Aldrich (Sintra, Portugal). (+)-catechin, phloridzin, procyanidin B1, quercetin-3-*O*-glucoside, quercetin-3-*O*-galactoside, luteolin-7-*O*-glucoside, kaempferol-3-*O*-glucoside, epicatechin gallate and protocatechiuc acid were supplied at Extrasynthese (Genay Cedex, France). Potassium persulfate and Folin-Ciocalteu from Merk (Algés, Portugal) and acetonitrile was obtained from Fisher Scientific (Oeiras, Portugal). 

### 2.2. Plant Materials

Fifteen natural extracts (Table 1) were selected for testing as browning inhibitors. In particular, strawberry tree leaves and branches wastes from pruning were provided by Medronhalva. Lda (central region of Portugal). Leaves were separated from branches and stored at −20 °C while branches were dried in a ventilated oven (70 °C) and stored at room temperature. Before extraction, leaves were freeze-dried and ground into a fine powder (IKA A10 analytical grinder) while dried branches were ground in a laboratory-scale mill. 

Apple byproduct, in particular pomace, was provided by INDUMAP-Fruit’s industrialization as a byproduct obtained from their production, Portugal, stored at −80 °C upon arrival, and used directly for extraction after thawed. 

### 2.3. Extraction Procedure

Each plant material (1:20 *m*/*v*) was consecutively extracted three times with methanol for 1 h under constant stirring at 25 °C with renewal of the solvent between extractions. The suspensions were then vacuum filtered, MeOH removed in a rotary evaporator (Büchi rotavapor R-114) and the extracts freeze-dried to obtain the final extract. Extraction yields obtained for strawberry tree leaves and branches were 44 and 11% respectively, while for apple byproduct it was 5.5%. After extraction, the optimal concentration, using deionized water as solvent, was determined considering solubility and color of each natural extract for seamless application. As it was intended to apply these extracts to fresh-cut pears it was crucial to ensure a concentration at which the extracts were total soluble and colorless without affecting the natural organoleptic characteristics of the fresh-cut pears (i.e. optimal concentration). A concentration of 9.5 mg mL^−1^, 5 mg mL^−1^ and 16 mg mL^−1^ was optimized for strawberry tree leaves, branches and apple byproduct, respectively.

### 2.4. Total Phenolic Content (TPC)

The TPC was determined as described elsewhere [16]. The reaction is based on the oxidation of polyphenols via Folin–Ciocalteu reagent, which is a mixture of phosphomolybdic and phosphotungstic acids, in a basic medium. The blue complex thus formed is assayed for absorbance at 750 nm, which is directly proportional to the total amount of polyphenols in the medium. To 0.05 mL of sample, 0.05 mL of Folin—Ciocalteu reagent, 1 mL of 75 mg mL^−1^ sodium carbonate and water were added up to the final volume of 2.5 mL. After 60 min in the dark at room temperature (23 °C), absorbance was measured (Thermo Scientific Multiskan GO). Results were compared with a standard curve prepared with gallic acid and reported as mg of gallic acid equivalent per g of dry weight of NES (mg GAE.g_NES_^−1^) ± sd of three replicates.

### 2.5. Antioxidant Activity by ABTS Radical Scavenging Activity Assay

The antioxidant capacity was assessed according to Oliveira et al. [17] using the radical scavenging assay with the ABTS^+^ radical. Briefly, ABTS radical cation solution was produced from the reaction of 7 mM 2,20- azinobis (3-ethylbenzothiazoline-6-sulfonic acid) diammonium salt (ABTS) and 2.45 mM potassium persulfate, after incubation, at room temperature (23 °C) in dark, for 16 h. After that, the ABTS solution was diluted with water to an absorbance of 0.70 ± 0.02 at 734 nm. After the addition of 1.0 mL diluted ABTS solution to 10 µL of the sample, the mixture absorbance reading was performed after 6 min. With the calibration curve, previously prepared with ascorbic acid as standard. The ABTS radical scavenging ability was calculated with this equation: [(A_0_- Asample)/A_0_] × 100 (% of inhibition), where A_0_ is the absorbance of the blank and Asample is the absorbance of the sample. The final result was expressed as the concentration of NES capable to scavenge 50% of the radicals (IC_50_) ± sd of three replicates.

### 2.6. In Vitro Polyphenoloxidase and Peroxidase Inhibition Assay

Prior to the NES inhibitory effect measurement on PPO and POX activity, the concentrations of enzyme, substrate and reaction time were optimized. The method was performed using a spectrophotometric assay measuring at 420 nm and 470 nm (Thermo Scientific Multiskan GO) the reaction of the natural-based extracts with the commercial enzymes, using catechol and guaiacol as substrates, respectively [18,19]. The enzyme activity in each inhibition reaction was determined using the SkanIt^TM^ Software version (4.1). The % of enzyme activity inhibition was calculated considering control (i.e., substrate without inhibitor) as the highest enzyme activity (i.e., 0% of inhibition). Ascorbic acid (1% m/v) was used as commercial inhibitors of PPO and POX (positive control).

### 2.7. Preparation and Treatment of Pear Disc

Pear fruit (*Pyrus communis L.cv* “Rocha”) were harvested from a 30-year-old commercial orchard in Cadaval Portugal. After harvesting, pears were immediately transported to a commercial packinghouse and hand-sorted to select undamaged fruit. Fruits were then stored at 4 °C until processing. Randomly, five-sliced “Rocha” pear discs (5 mm thick, 15 mm diameter) from nine fruits (i.e., a total of 5 discs per treatment) were prepared and sprayed immediately with NES at the optimal concentration (9.5 mg mL^−1^, 5 mg mL^−1^ and 16 mg mL^−1^ for strawberry tree leaves, branches and apple byproduct, respectively). All the extracts were filter sterilized (0.22 mm) before application to avoid contaminations. After treatment, pear discs were kept at room temperature (23 °C; 66% RH), exposed to air for the monitorization of browning appearance. According to Bajwa et al. [4] browning was measured using CR-400 Chroma meter (Konica Minolta Sensing Americas Inc., NJ, USA) in the first 15 min and then every 2 h interval during 6 h. The changes in tissue lightness, (ΔL = L_initial_ − L) was used to quantify the browning of pear discs, where L_initial_ represents the tissue lightness at time-point 0 h and L at each time-point (2, 4 and 6 h). Cut-pear discs treated with water were used as experimental control and ascorbic acid (1% *m*/*v*) as commercial control (positive control). 

### 2.8. Identification of Phenolic Compounds by LC-ESI-UHR-QqTOF-HRMS

NES were dissolved in methanol, filtered and analyzed by Liquid-Chromatography- Electrospray-Ionization-Quadrupole-Time-of-Flight-High-Resolution-Mass-Spectrometry (LC-ESI-UHR-QqTOF-MS) according to Monforte et al. [20]. The separation was performed in a UHPLC UltiMate 3000 Dionex (Thermo Scientific), coupled to an ultrahigh-resolution. Qq-time-of-flight (UHR-QqTOF) mass spectrometer with 50,000 full-sensitivity resolution (FSR) (Impact II, Bruker Daltonics, Bremen, Germany). Separation of metabolites was performed using an Acclaim RSLC 120 C18 column (100 mm × 2.1 mm, 2.2 μm) (Dionex). Mobile phases were 0.1% aqueous formic acid (solvent A) and acetonitrile with 0.1% formic acid (solvent B). Separation was carried out over 24.5 min under the following gradient conditions: 0 min, 0% B; 10 min, 21.0% B; 14 min, 27% B; 18.30 min, 58% B; 20.0 min, 100%; 24.0 min, 100%; 24.5 min, 0% B at a flow rate of 0.25 mL min^−1^. The injection volume was 5 μL. Parameters for MS analysis were set using negative ionization mode with spectra acquired over a range from *m*/*z* 20 to 1000. The parameters were as follows: capillary voltage, 3.0 kV; drying gas temperature, 200 °C; drying gas flow, 8.0 L.min^−1^; nebulizing gas pressure, 2 bar; collision RF, 300 Vpp; transfer time, 120 μs; and prepulse storage, 4 μs. Post-acquisition internal mass calibration used sodium formate clusters with the sodium formate delivered by a syringe pump at the start of each chromatographic analysis. High-resolution mass spectrometry was used to identify the compounds. The elemental composition for the compound was confirmed according to accurate mass and isotope rate calculations designated mSigma (Bruker Daltonics). The accurate mass measurement was within 5 mDa of the assigned elemental composition, and mSigma values of <20 provided confirmation. Compounds were identified based on its accurate mass [M-H]^−^. 

### 2.9. Quantification of Phenolic Compounds by HPLC-PDA

The quantitative profiles of phenolic compounds in NES were performed using a Waters Alliance e2695 (Waters, Mildford, MA, USA) separation module system interfaced with a photodiode array UV/Vis detector 2998 (Waters, Mildford, MA, USA). The separation of the compounds was carried out in a reverse phase C18 column (ZORBAX Eclipse XDB-C18 Packed Column—4.6 mm I.D. × 250 mm; Agilent, Santa Clara, CA, USA). The mobile phases and the gradient program used were prepared according to Vilas-Boas et al. [21] with some modifications. The mobile phase was composed of solvent A: water/acetonitrile/TFA (94.9/5/0.1) and solvent B: acetonitrile/TFA (99.9/0.1) with the elution gradient: 0–1 min 0% B; 1–30 min 21% B; 30–42 min 27% B; 45–55 min 58% B; 55–60 min 0% B and kept another 1 min a 0% B. Flow rate was 1 mL min^−1^, the oven temperature was set at 25 °C and the injection volume was 20 μL. Detection was performed at 280 nm, 320 nm and 350 nm, while data acquisition and analysis were accomplished using Software Empower 3. Retention times and spectra of the identified were analyzed by comparison with pure standards (arbutin, gallic acid, caffeic acid, ellagic acid, rutin, syringic acid, (+)-catechin, phloridzin, quercetin 3-glucoside, quercetin 3-galactoside, luteolin-7-*O*-glucoside, kaempferol 3-*O*-glucoside, epicatechin gallate and protocatechiuc acid). Only the identified compound and their derivatives with pure standards commercially available were quantified. Herein, procyanidin dimer B type was calculated as equivalents of procyanidin B1, kaempferol derivate as equivalents of kaempferol, quercetin derivates as quercetin-3-*O*-glucoside equivalent and gallic acid derivates as equivalents of gallic acid. The other compounds were quantified using the pure standard curve. The quantification was performed by the calibration curves and the results were expressed as µg per mg of dry weight of NES (µg mg_NES_^−1^) ± sd of three replicates.

### 2.10. Statistical Analysis

The concentration giving 50% inhibition of both antioxidant activity and enzymatic inhibition (IC_50_) was calculated by nonlinear regression with the use of Prism GraphPad Prism, version 8.0 for Windows. Additionally, the principal component analysis (PCA) using Statgraphics Centurion XVII software was performed to the data set, using the mean value of each analysis, after normalization and factor analysis was performed to reduce and explain the variability of the data. The Varimax method was used to produce orthogonal transformations to the reduced factors as to better identify the high and low correlations. One-way analysis of variance (ANOVA) test followed by multi-comparisons Fisher’s Least Significant Difference (LSD) post-hoc test was applied to determine significant differences between NES. Differences with a probability value of *p* < 0.05 were considered significant and all data were reported as mean ± sd.

## 3. Results and Discussion

### 3.1. Chemical Characterization of NES

Fifteen natural extracts (Table 1) were tested as browning inhibitors. Firstly, the optimal concentration was scrutinized, considering solubility and color of each natural extract for seamless application. Then, the antioxidant activity and tissue browning inhibition of all the natural extracts were assessed (data not shown). Based on this screening, the selected extracts (NES) were strawberry tree (leaves and branches) and apple byproduct. A concentration of 9.5 mg mL^−1^, 5 mg mL^−1^ and 16 mg mL^−1^ was used for strawberry tree leaves, branches and apple byproduct, respectively. NES were further investigated regarding their in vitro biological activities and composition. Moreover, to the best of our knowledge, the information about strawberry tree branches antioxidant activity and total phenolic content is reported for the first time. 

#### 3.1.1. Total Phenolic Content

Various extraction methods have been provided from literature, but studies on strawberry tree indicated that phenolic compounds are efficiently extracted using alcoholic solutions [22,23]. Thus, in this work, all the extractions were performed following this approach. Owing to their protective effects, phenolic compounds have sparked vast interest in several biological research fields. Phenolic compounds are plants’ natural constituents involved in their protection mechanism through different biological functions, including metal chelation, ROS neutralization, and of particular relevance in this study, its antioxidant action [24].

The TPC of NES is presented in Table 2. Data revealed that TPC varied significantly among NES. It is clear from the presented data that the organic extracts of strawberry tree have the highest TPC, despite the significant difference found in both parts used. Chiocchio et al. [25] also demonstrated the natural phytochemical variability between parts of the same plant material. Leaves extract showed the highest TPC (201.97 ± 18.05 mg GAE.g_NES_^−1^) (*p* < 0.05), being 2-fold higher than that found in branches extract (104.07 ± 16.38 mg GAE.g_NES_^−1^). Malheiro et al. [26] have already reported the bioactive properties of strawberry tree of great interest for chemical and food industries. Indeed, our results regarding strawberry tree leaves extract are in agreement with those reported by Moualek et al. [27], which showed that strawberry tree leaves (from Tizi-Ouzou, Algeria) extracted with water for 24 h, presented 207.84 ± 15.03 mg GAE.g_extract_^−1^. Recently, Erkekoglou et al. [28] found a similar TPC (213.8 ± 3.2 mg GAE.g_extract_^−1^) in strawberry tree leaves extract, collected in Greece and extracted with hot water. Yet, the TPC of the extracts used in the current study is higher than reported by Oliveira et al. [29] using ethanol extracts of strawberry tree leaves, collected in Bragança (the northeastern region of Portugal), with 172.21 ± 6.29 mg GAE. g_extract_^−1^. Bouyahya et al. [30] and Pavlović et al. [31] obtained lower values of TPC using strawberry tree leaves collected in Morroco and Montenegro, respectively and obtained via solid-liquid and hydroalcoholic percolation, respectively. In recent work, Tenuta et al. [32] presented a TPC of 173.33 ± 1.20 mg chlorogenic acid equivalents.g_dry extract_^−1^ of a hydroalcoholic extraction of strawberry tree leaves from Southern Italy. In general the strawberry leaves extract used in this study have a higher TPC content than those reported before [26,27,28,29,30,31,32]. This phytochemical intra-variability could be related to slightly different edaphoclimatic conditions of the different locations and extraction procedure [26]. The age of the plant and the time of the year to collect leaves and branches could be a factor to support the variability observed [33].

The TPC of apple byproduct was revealed to be much lower than strawberry tree extracts (6.76 ± 0.11 mg GAE.g_NES_^−1^) (*p*< 0.05). Since this byproduct is obtained from industrial processing, the lower TPC could be explained by its vulnerability to phenolic compound loss, mainly due to oxidation reactions. Similar results were obtained by Du et al. [34] where a TPC of 0.208 mg GAE.mL^−1^ of apple pomace was obtained. 

#### 3.1.2. Antioxidant Activity

**Figure 1:**
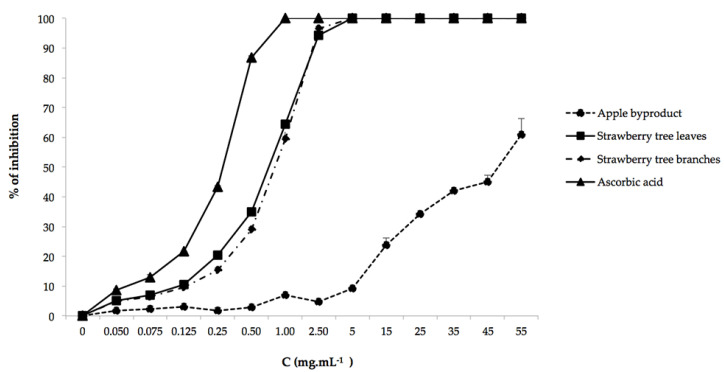
Values of ABTS^+^ radical inhibition (%) by NES. Ascorbic acid was used as positive control. Data are mean ± standard deviation (SD) (n = 3).

The ABTS method is widely used as a simple method, which permits a comprehensive assessment of the antioxidant potential of extracts in a short time interval [35]. The parameter used to compare the radical scavenging potential of the extracts was the IC_50_ value, which represents the concentration of antioxidant required for scavenging 50% of ABTS radicals. So, the lower the IC_50_ value, the higher the antioxidant activity power of the extracts. In order to evaluate the antioxidant efficiency of NES, their IC_50_ values were compared with 1% ascorbic acid, as the positive control, since it is one of the most potent antioxidants used in food formulations IC_50_ (0.25 ± 0.08 mg mL^−1^). In Figure 1, a concentration-dependent activity for reducing power is observed. Although NES IC_50_ was significantly higher than ascorbic acid (Table 2), high values of reducing power were obtained with strawberry tree leaves and branches (IC_50_ = 0.65 ± 0.11 mg mL^−1^ and 0.75 ± 0.06 mg mL^−1^, respectively). It is clear that the strawberry tree extracts showed the highest radical scavenging activity and therefore the highest antioxidant activity, which is coherent with the higher TPC reported above. Moreover, this behavior has been also reported by Mokrani et al. [36], who demonstrated a good correlation between TPC and the antioxidant capacities of peach fruit extracts, indicating that phenolic compounds are major contributors to their antioxidant capacity. Thus, the results obtained suggest that strawberry tree extracts may react with free radicals, converting them to more stable products [29,37]. 

As expected, strawberry tree leaves extract demonstrated to be much more effective, following the same behavior of TPC analysis. Similar results were obtained by Mendes et al. [37] where strawberry tree leaves extracts exhibited the best antioxidant activity among the other natural extracts tested. Also, high values of reducing powers were achieved at low concentrations (IC_50_ values < 0.65 mg mL^−1^) by Malheiro et al. [26]. Moreover, it must be noted that synergic effects between different antioxidant components are often important in the overall antioxidant activity. Cooperative action between phenolic compounds and other antioxidants has also been reported by Stahl and Sies [38] and, therefore, the differences of antioxidant activity between strawberry tree extracts may putatively reflect this combined action. 

Apple byproduct showed the highest IC_50_ (45.59 ± 1.34 mg mL^−1^) and, therefore, the lowest antioxidant activity. This low antioxidant activity could be due to the antioxidants losses promoted by industrial processing. The effectiveness of NES as antioxidants is significantly affected by their phenolic composition and number of hydroxyl groups, which could explain the differences observed between them [39]. This is further investigated through the characterization of the phenolic compounds of NES. 

#### 3.1.3. Characterization of Phenolic Compounds Composition of NES

Regarding the qualitative phenolic composition of NES, extracts were analyzed by LC-ESI-UHR-QqTOF-MS (Table 3) and, as expected, a great variety of phenolic compounds were identified based on their mass spectral characteristics and MS fragmentation. However, only a few of these phenolic compounds were subsequently quantified by HPLC-PDA in the different NES (Table 4). The phytochemicals profile of NES showed the presence of hydroxycinnamic, hydroxybenzoic acids and phenolic glucosides, flavonols, and flavanols, as the main classes of constituents. The broad variety of phenolics found in strawberry tree leaves, which justified the high TPC measured and antioxidant activity measured, has already been reported in the literature [26,29,30,32,37,40,41,42,43]. Among them, the most worth mentioning components are arbutin, along with catechin [40], which is consistent with the quantitative results obtained in the present study (52.33 ± 0.61 and 10.18 ± 0.25 µg mg^−1^_NES_, respectively). 

Leaves are also characterized by the presence of gallic, syringic, and caffeic acids [26,29,30,32,37,40,41,42,43]. In fact, gallic (3.52 ± 0.32 µg mg^−1^_NES_) and syringic acids (0.65 ± 0.36 µg mg^−1^_NES_) were extracted, while caffeic acid was not detected in the present study. Recently, Tenuta el al. [32] also confirmed the presence of these phenolic acids in strawberry tree leaves extracts obtained via hydroalcoholic extraction. These phenolic compounds have been associated with a wide range of biological activities in foods, including antioxidant, antimicrobial, prevention of enzymatic browning, between others [41,44]. In particular, arbutin was also reported as a whitening agent because of its antioxidant and free radical scavenging properties [45]. These phenolic acids possess structural features that trigger the H-atom donation resulting in antioxidant scavenging capacity [44]. Luteolin 7-*O*-glucoside, a very common flavonol in leaves, was also found in relevant amounts (7.37 ± 0.01 µg mg^−1^_NES_) [46]. This flavonol is known for the 2,3-double bond conjugated with a 4-oxo function, which is responsible for electron delocation between the flavonoid rings contributing to more antioxidant activity [46]. 

In literature, leaves have been investigated more than the branches [47,48]. Moreover, to the best of our knowledge, the information about the biological activities and the phytochemical contents of branches extracts is scarce. Dib et al. [43] identified (+)-catechin, (+)-afzelechin and (+)-2-(3,4-dihydroxyphenyl)-5,7-dihydroxy chroman- 3-yl-4-hydroxybenzoate. A lower amount of arbutin (7.43 ± 1.14 µg mg^−1^_NES_) in strawberry tree branches was observed (7 times lower) compared to strawberry tree leaves extracts. Moreover, the natural-based antioxidant extract from the branches showed more quantity of procyanidin B type than the extract from the leaves. Regarding the flavonols class, the extracts from branches showed higher contents of kaempeferol-3-*O*-glucoside (2.17 ± 0.02 µg mg^−1^_NES_), quercetin-3-*O*-glucoside (0.55 ± 0.03 µg mg^−1^_NES_) and quercetin derivates than in leaves extract. This group is characterized by a higher number of hydroxyl groups, which is also responsible for high antioxidant capacity. They are also known as potent inhibitors of several enzymes, such as xanthine oxidase, cyclo-oxygenase, lipoxygenase, and phosphoinositide 3-kinase [49]. Proanthocyanidins were also detected at high concentrations in strawberry tree branches. Additionally, higher concentration of gallic acid (4.85 ± 0.17 µg mg^−1^_NES_) and syringic acid (1.24 ± 0.07 µg mg^−1^_NES_) were obtained.

Regarding apple byproduct extract, several authors have reported its phenolic compounds, exhibiting as main components (-)-epicatechin, chlorogenic acid, caffeic acid, quercetin, *p*-coumaric acid, and phloridzin as the main compounds [50,51,52,53,54]. However, since this byproduct is obtained from industrial processing of apples from different varieties and origins, higher variability is found between different apple byproduct extracts. The lower phenolic content observed, especially in phenolic acids (Table 3 and Table 4) could be explained by its vulnerability to oxidation reactions throughout its storage. In this study, phenolic compounds contents found in apple byproducts are quite lower, compared to strawberry tree extracts, which corroborates the lower TPC and antioxidant activity previously obtained (Table 2). However, phloridzin was only detected in this extract (0.51 ± 0.01 µg mg^−1^_NES_). Phloridzin is a phenolic compound that occurs naturally in apples and is associated with several health-promoting effects [55]. Furthermore, phloridzin has been identified as a potent antioxidant, particularly in inhibiting lipid peroxidation [56]. Moreover, Wang et al. [57], demonstrated the inhibitory effect of phloridzin on the activity of mushroom tyrosinase, also known as mushroom PPO. Therefore, although this extract presents little diversity and quantity of phenolic compounds, it presents a dihydrochalcone with great different bioactivities reported. 

### 3.2. Inhibitory Effect of the Selected Extracts on Browning Enzymes Acitivity

The potential of the extracts in reducing or blocking the enzymes activity responsible for browning in vitro, PPO and POX, is of high interest. The effectiveness of NES in restraining these enzymes’ activity was examined using solutions containing the respective substrate and NES. The inhibition was plotted against NES concentration to calculate the concentration that caused 50% activity reduction (IC_50_). According to Table 5, which represents the activity against PPO and POX, the IC_50_ values calculated indicate that none of the extracts was as effective as ascorbic acid, with strawberry tree extracts being the most potent (*p* < 0.05). However, strawberry tree leaves extract was expected to have a higher effect against PPO due to its higher antioxidant activity. Yet, despite the results of PPO inhibition, the extracts seem to be quite effective in reducing POX activity. Both strawberry tree extracts were able to block POX at lower concentrations. In particular, strawberry tree leaves extract was able to inhibit 50% of the enzyme activity at a concentration similar to ascorbic acid. Once more, the results emphasize the possibility of using strawberry tree extracts as browning inhibitors, despite the different parts of the plant which was expected after the range of phenolic compounds, especially phenolic acids and flavonols found in their composition. These enzymes accept several polyphenols as substrates, thus the potential inhibitory role of NES may depends on their phenolic compounds composition [58]. Thus, it is important to highlight the relatively marked inhibitory effect of strawberry tree extracts, probably indicating the presence of phenolic compounds similar to the enzyme’s substrate (i.e., with analogous affinity to the active site of the enzymes). These can act through a competitive mechanism or bind to the enzyme, thus affecting the catalytic reaction of PPO and POX [59]. Various phenolic compounds, such as procyanidins, quercetin, quercetin-glucoside, and rutin have been described for their ability to bind proteins [39,60,61]. Le Bourvellec et al. showed that procyanidins, flavanol polymers that appear in plants, inhibited PPO activity in cider apple juices [62]. Also, it is noteworthy that the enzymatic inhibition could be associated with the presence of compounds with multiple phenolic OH groups [63]. 

Despite the high content of arbutin (a strong anti-browning component [25]) in strawberry tree extract, the higher IC_50_ found, compared to ascorbic acid, suggests that the presence of catechin (a pro-oxidant agent) could be serving as a substrate for the enzymes, increasing its activity [64,65].

The lower content of phenolic compounds in apple byproducts extracts explains the higher IC_50_ values against browning enzymes. Chiocchio et al. [25], found a linear correlation between enzymatic activity and TPC, which is in agreement with the results obtained, i.e., the higher the phenolic content the lower IC_50_. After the measurement of the in vitro potential of NES in restraining the enzymes associated with browning, it was crucial to understanding their effect in situ. For this reason, the inhibitory effect of NES in avoiding browning in fresh-cut pear was measured. 

### 3.3. Efficacy of the Natural Extracts in Inhibiting Browning in Pear Cut Discs

**Figure 2:**
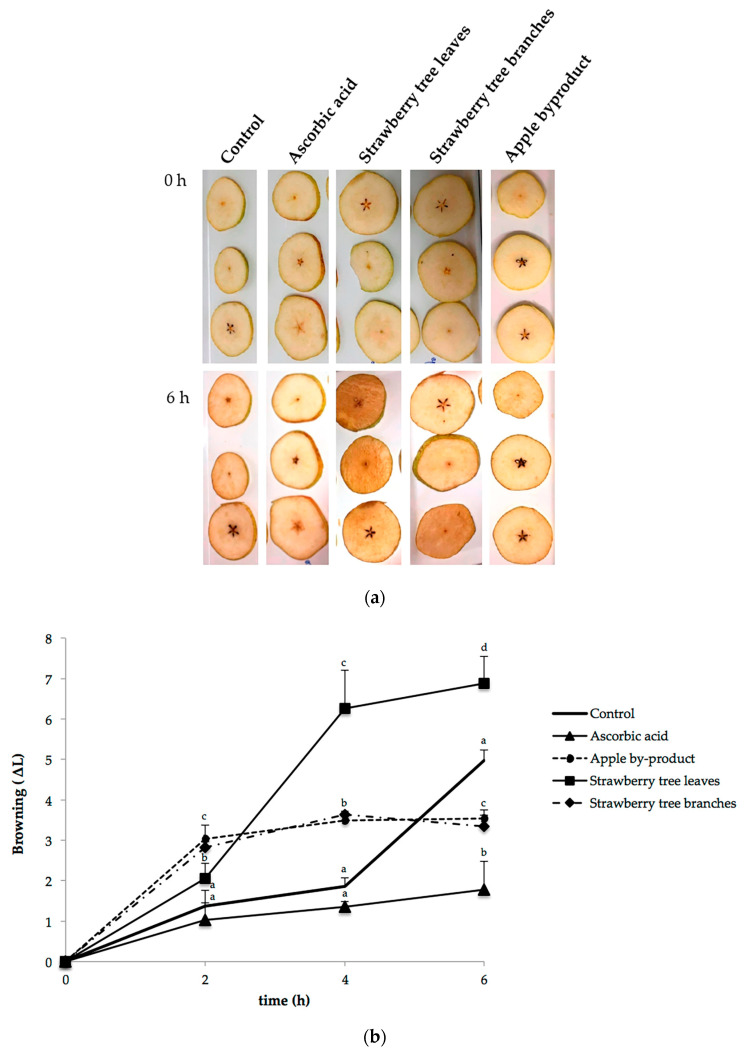
Results of fresch-cut pear fruits browning inhibition by NES: (**a**) Pear discs dipped in different NES solutions NES (discs sprayed with water and ascorbic acid were used as control and positive control, respectively) during 6 h. (**b**) The y-axis shows the color change (∆L) of the treated discs, measured every 2 h interval during 6 h of exposure to air. Data are mean ± standard deviation (SD) (n = 5). Different superscript letters differ significantly by LSD test (*p* < 0.05).

The visual examination of “Rocha” pear discs sprayed with NES, water (control) and ascorbic acid (commercial/positive control), and exposed to air for 6 h, are represented in Figure 2. The variations in tissue lightness (Figure 2b) are coincident with the manifestation of browning (Figure 2a). In fact, Soliva-Fortuny et al. [66] demonstrated the postitive correlation between L* and browning. After 2 h, all conditions suffered an increase in browning tissue. The observed increase in ΔL* values in all conditions at the first 2 h, can be explained by the fact that PPO and POX are very active initially, as a wounding injury stress reaction. Similar effects were observed in fresh-cut chestnut and peach slices [67,68]. In this way, both enzymes and intrinsic phenolic compounds come into contact and react leading to browning appearance [69]. Bachra et al. [59] demonstrated that, in tomato, PPO was wound-induced. Also, the rise in some phenolic substrates is related to the wounding damage that rises secondary metabolite production related to browning [70]. Figure 2b also discloses that ΔL* values for pear discs treated with strawberry tree leaves extracts increased with time after treatment. A possible explanation for the sharp increase of ΔL* values in cut pear discs treated with strawberry tree leaves extracts could be the lack of penetration of the extract into the cellular matrix darkening the surface, because of the color of the extract, or the oxidation of the pro-oxidant phenolic compound present in the extract by PPO and POX [71,72]. For example, catechins, one of the phenolic compounds found in strawberry tree leaves extract, has been found to contribute to enzymatic browning of foods [64,65]. Also, since optimized concentrations were used based on color and solubility, it is important to have in mind that browning inhibition reactions are concentration-dependent [73]. In opposition to control behavior, no differences were found in ΔL* values from 2 until 6 h for strawberry tree branches and apple byproduct extracts. One of the most applied agents to reduce browning of fresh-cut fruit surfaces is ascorbic acid [74]. As expected, during the exposure extend, the ascorbic acid treatment effectively retarded cortex-browning appearance and was the most effective in controlling browning. Interestingly, similar behavior to ascorbic acid was obtained with apple byproduct extracts. It is important to highlight that the unchanged ΔL* values can indicate protection against browning. Wang et al. [57], reported the inhibitory effects of phloridzin on PPO activity. Phloridzin, one of the polyphenols identified in apple byproduct, was able to inhibit the diphenolase activity of PPO in a competitive manner [57]. Yet, it was observed a browning inhibition by apple byproduct when applied in situ to fresh-cut pears. The contrary results obtained in vitro, high IC50 (lower anti-browning potential), may be caused by differences between enzyme activity in vitro and in situ, as stated for other enzymes [75]. Also, a correlation between browning and enzyme activity is not always observed [76]. There are matrix-related issues, such as membrane integrity, and interactions that do not occur in vitro and could explained the results obtained in vitro and in situ [76]. Moreover, it must be noted that synergies between different antioxidant components are not being taken into account and is likely important in the browning inhibition observed in situ, which could be also an explanation for the different results obtained in vitro and in situ [37]. A similar effect was obtained for strawberry tree branches extract. This is in line with the results obtained in vitro. Hence, the inactivation of the oxidative enzymes due to an intrinsic synergy between the biological compounds of NES can be one of the reasons that supports NES anti-browning properties [9,77].

### 3.4. Principal Component Analysis

**Figure 3:**
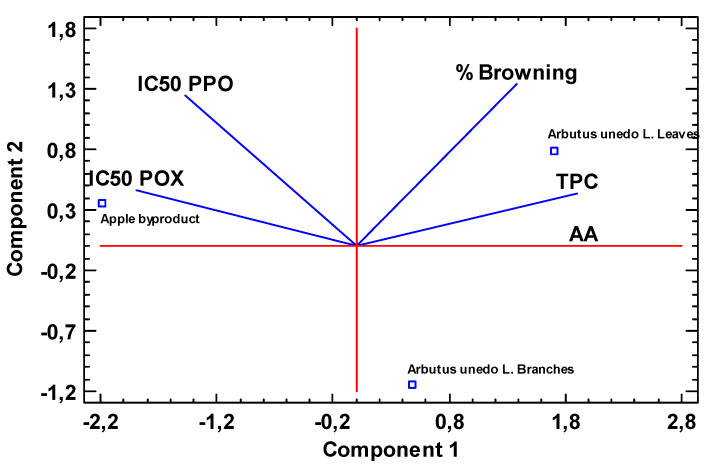
PCA analysis for % Browning, TPC, AA, IC50 PPO, and IC50 POX. The first component explained 79.24% of the variance across the extracts and the second component explained 20.77% of the variance.

As a summary and for a better overview between the different NES, a PCA analysis (Figure 3) was executed to support the differences between NES from different sources regarding the parameters TPC, AA, % of browning, and oxidative enzymes IC_50_. PCA biplot shows the information about samples (dots) and variables (vectors) as a data matrix. Component 1, which describes 79.24% of parameters variability, was positively influenced by three variables (% Browning, TPC, and AA), whereas, component 2 describes 20.77% of the variability. As aforementioned, TPC and AA are highly correlated (*R*^2^ = 0.999) and it is shown in the PCA analysis, since they are coupled in the positive side of PC1. Strawberry tree leaves extract are represented in the positive values of PC1, which confirmed their higher phenolic content and antioxidant potential and higher % browning appearance in fresh-cut pears treated with strawberry tree leaves extracts. Additionally, the IC_50_ value for both enzymes (PPO and POX) is represented in the negative side of PC1 indicating the lower values obtained for this extract. Strawberry tree branches extract is represented in the positive values of PC1 and negative values of PC2, because it showed promising results of % browning, TPC and AA (lower than strawberry tree leaves extract but higher than apple byproduct extract) and PPO and POX IC_50_. Despite being in the negative values of PC1, highlighting it’s lower antioxidant and phenolic content, apple byproduct is represented in the opposite site of % browning, demonstrating its potential in reducing tissue browning, even with low content of TPC and AA. It is also observed that this extract is on the same side of IC_50_ parameter of both enzymes, which indicates the higher values (less inhibitory effect against PPO and POX in vitro). This PCA analysis also validates the phenolic characterization of NES, confirming the variety of phenolic compounds found in strawberry tree extracts compared to apply byproduct.

## 4. Conclusions

The present study allowed us to demonstrate the biological activity and anti-browning potential of three natural-based methanolic extracts. The record of total phenolic content and antioxidant activity was crucial to infer the potential of strawberry leaves and branches as valuable sources of bioactive compounds to be applied as potential browning mitigators. Yet, these extracts exhibited high phytochemical variability using distinctive plant parts. Moreover, despite the evidence of extracts PPO inhibition effect, PPO activity was noticeably lower than POX. Overall, these extracts have stood out as prosperous natural alternatives on fresh-cut fruit browning inhibition. Apple byproduct extract used in this study, in particular apple pomace obtained from industrial apple processing, was revealed to be less effective in reducing the activities of the oxidative enzymes, however, this byproduct showed potential in delaying fresh-cut pear browning expansion. The effectiveness of extracts as enzymatic inhibitors is related to their enriched phenolic compounds. The present study has practical implications in the development of novel natural extracts with potential applications as anti-browning agents. Further biochemical investigation about NES mode of action in situ is needed as well as to determine cytotoxicity, nutritional profiling studies, sensory properties of all NES are warranted.

## Figures and Tables

**Table 1:** Listof all the natural sources screened in this study for potential anti-browning effect.

Plant Name and Part	
Elderberry flower (*Sambucus* L.)	Vine (*Vitis vinifera* L.) leaves
Pear (*Pyrus communis* L. “Rocha”) pulp	Vine (*Vitis vinifera* L.) branches
Pear (*Pyrus communis* L. “Rocha”) peel	Olive (*Oleo europaea* L.) leaves
Pear (*Pyrus communis* L. “Rocha”) pomace	Olive (*Oleo europaea* L.) branches
Apple (*Malus domestica* L.) peel	Acorn (*Quercus* L.) bark
Apple (*Malus domestica* L.) pomace	Bitter Melon (*Momordica charantia* L.) whole plant
Strawberry tree (*Arbutus Unedo* L.) leaves	Potato plant (*S.tuberosum* L.) leaves
Strawberry tree (*Arbutus Unedo* L.) branches	

**Table 2:** Total phenolic content (TPC) and Antioxidant activity of natural-based extracts (NES). Ascrobic acid is the positive control.

NES	Total Phenolic Content	Antioxidant Activity (AA)
	TPC	IC_50_
Strawberry tree leaves	207.97 ± 0.007 ^b^	0.65 ± 0.11 ^b^
Strawberry tree branches	104.07 ± 16.38 ^c^	0.75 ± 0.06 ^c^
Apple byproduct	6.76 ± 0.11 ^d^	45.59 ± 1.34 ^d^
**Positive control**		
Ascorbic acid	938.35 ± 18.05 ^a^	0.25 ± 0.08 ^a^

AA = IC_50_ (mg mL^−1^); TPC = mg of gallic acid equivalent per g of NES (mg GAE.g_NES_^−1^), in the case of ascorbic acid (positive control) TPC = mg GAE.g_ascorbic_ acid. Data are mean ± standard deviation SD (n = 3). Different superscript letters differ significantly by Fisher’s Least Significant Difference LSD test (*p* < 0.05).

**Table 3:** LC-ESI-UHR-QqTOF-MS data for phenolic compounds identified in NES.

Postulate Compounds	Rt	[M-H]^−^	MS^2^ Ions (*m*/*z*)	Molecular	Strawberry Tree	Strawberry Tree	Apple
	(min)	(*m*/*z*)		Formula	Leaves	Branches	Byproduct
***Hydroxybenzoic acids***							
1-O-Vanilloyl-β-D-glucose	8.8	329.09	167	C_14_H_17_O_9_	√	√	√
Vanillyl alcohol	6.7	153.06	123	C_8_H_9_O_3_	nd	nd	√
Gentisic acid	6.5	153.02	109	C_7_H_5_O_4_	nd	√	nd
Protocatechiuc acid	6.2	315.07	108, 152	C_13_H_15_O_9_	nd	√	nd
Protocatechualdehyde	5.6	137.02	93	C_7_H_5_O_3_	nd	nd	√
Gentisic acid 5-*O*-β-glucoside	6.2	315.06	108, 151	C_13_H_15_O_9_	√	nd	√
Gentisic acid 2-*O*-β-glucoside	5.6	315.07	109, 153	C_13_H_15_O_9_	nd	nd	√
Gentisic acid derivative	6.5	315.11	153	C_13_H_19_O_8_	√	nd	nd
4-Hydroxybenzoic acid	5.6	137.02	93	C_7_H_5_O_3_	√	nd	nd
4-Hydroxybenzoate-β-D-glucoside	5.4	299.08	9, 137	C_13_H_15_O_8_	√	nd	nd
4-Hydroxybenzoate-β-D-glucoside	6.4	299.08	59, 137, 179	C_13_H_15_O_8_	√	nd	nd
1-O-Galloyl- β-D-glucose	8.9	577.13	289, 407, 425	C_13_H_15_O_10_	√	√	nd
Gallic acid	13.1	433.08	300	C_7_H_5_O_5_	√	√	nd
Digallic acid	8.5	321.03	169	C_14_H_9_O_9_	nd	√	nd
4-O-Galloylshikimic acid	12.3	939.11	769	C_14_H_13_O_9_	√	√	nd
Syringic acid	7.6	577.14	289, 407	C_9_H_15_O_7_	√	√	nd
***Hydroxycinnamic acids***							
*p*-Coumaric acid	8.5	163.04	119	C_9_H_8_O_3_	√	√	√
*p*-Coumaroyl-β-D-glucose	8.5	325.10	119, 163	C_15_H_17_O_8_	√	√	√
Caffeoylquinic acid	8.4	353.12	191.05	C_16_H_18_O_9_	nd	nd	√
1,3 dicaffeoylquinic acid	13.2	515.12	191	C_25_H_23_O_12_	nd	√	nd
Caffeic acid 3-β−D-glucoside	8.2	341.09	135, 179	C_15_H_17_O_9_	nd	nd	√
3,4-Caffeoyl alcohol-O-hexoside	7.9	327.11	165	C_15_H_19_O_8_	nd	nd	√
1-O-Feruloyl-β-D-glucose	9.2	355.10	175, 193	C_16_H_19_O_9_	nd	nd	√
1-O-Feruloyl-β-D-glucose	10.2	355.10	175, 193, 235	C_16_H_19_O_9_	nd	nd	√
***Flavones***							
Isoorientin 2″-O-rhamnoside	12.8	593.1512	285	C_27_H_29_O_15_	√	√	nd
2″-O-Galloylorientin	12.9	599.1042	169, 313, 447	C_28_H_23_O_15_	√	nd	nd
Isoorientin 6″-O-p-hydroxybenzoate	15.8	567.1144	284	C_28_H_23_O_13_	√	nd	nd
***Flavanols***							
Procyanidin dimer B type	8.9	577.13	289, 407, 425	C_30_H_26_O_12_	√	√	√
Ellagic acid 4-α-L-arabinofuranoside	13.1	433.08	300	C_19_H_14_O_12_	nd	nd	√
Penta-O-galloyl-β−D-glucose	12.3	939.11	769	C_41_H_31_O_26_	√	√	nd
Procyanidin B5	7.6	577.14	289, 407	C_30_H_25_O_12_	√	√	nd
Procyanidin B4	7.9	577.14	125, 289, 407	C_30_H_25_O_122_	√	√	nd
Procyanidin B-5,3′-O-gallate	9.7	729.15	289, 407, 577	C_34_H_29_O_16_	√	√	nd
Procyanidin B-13′-O-gallate	10.3	729.15	289, 407, 577	C_37_H_29_O_16_	√	√	nd
(-)-Epicatechin gallate	11.8	441.08	169, 289	C_22_H_17_O_10_	√	√	nd
(+)-Catechin	8.3	289.07	109, 123	C_15_H_13_O_6_	√	√	nd
***Flavonols***	13.4	447.09	300, 301				
Kaempferol 3-*O*-β−D-glucoside	12	463.09	300	C_21_H_19_O_11_	√	√	√
Quercetin 3-*O*-β−D-glucoside	12.2	463.09	300	C_21_H_19_O_12_	√	√	√
Quercetin 3-*O*-β−D-galactoside	12.8	593.15	285	C_21_H_19_O_12_	√	√	√
Kaempferol-3-*O*-β−D-rutinoside	13.4	447.09	300, 301	C_27_H_29_O_15_	nd	nd	√
Kaempferol 3-*O*-β−D-xyloside	14.4	417.08	284	C_20_H_17_O_10_	√	√	nd
Kaempferol 3-*O*-α L-rhamnoside	14.8	431.10	285	C_21_H_20_O_10_	√	nd	nd
Luteolin 7-*O*-β−D-glucoside	13.4	447.09	300, 301	C_21_H_19_O_11_	√	nd	nd
Quercetin derivative		433.08		C_20_H_17_O_11_	√	√	nd
Quercetin hexose galloyl derivative	11.3	615.10	300, 463	C_28_H_23_O_16_	√	√	nd
Myricetin 3-O-α−D-rhamnoside	11.8	463.09	316	C_21_H_19_O_12_	√	√	nd
Rutin	11.9	609.15	300, 301	C_27_H_29_O_16_	√	√	nd
8-Hydroxykaempferol	16.7	301.04	121, 151, 178	C_15_H_9_O_7_	√	√	nd
Quercitrin-2″-gallate	13.1	599.10	169, 313, 463	C_28_H_23_O_16_	nd	√	nd
***Other Flavonoids***							
Taxifolin	8.6	303.05	125, 153	C_15_H_11_O_7_	√	√	nd
Taxifolin	13.6	303.05	151	C_15_H_11_O_7_	√	√	nd
***Chalcone***							
Arbutin	2.1	271.08	108	C_12_H_15_O_7_	√	√	nd
***Dihydrochalcones***							
Phloretin glucoside	13.2	567.17	273, 167	C_26_H_31_O_14_	nd	nd	√
Phloridzin	14.5	435.13	273	C_21_H_23_O_10_	nd	nd	√

Rt: Retention time; √—detection; nd—not detected.

**Table 4:** Concentration of phenolic compound present in NES.

Postulate Compounds	Strawberry Tree	Strawberry Tree	Apple
	Leaves	Branches	Byproduct
***Hydroxybenzoic acids***			
Protocatechiuc acid	nd	1.15 ± 0.01	0.05 ± 0.01
***Flavanols***			
Procyanidin dimer B type	8.78 ± 0.13	6.01 ± 1.10	nd
Procyanidin dimer B type	nd	5.53 ± 1.31	nd
(-)-Epicatechin gallate	2.76 ± 0.09	1.79 ± 0.07	nd
(+)-Catechin	10.18 ± 0.25	12.38 ± 1.21	nd
***Flavonols***			
Kaempferol 3-*O*-β−D-glucoside	0.53 ± 0.01	2.17 ± 0.02	0.10 ± 0.001
Kaempferol derivate	9.90 ± 0.02	nd	nd
Kaempferol derivate	0.58 ± 0.004	nd	nd
Quercetin 3-*O*-β−D-glucoside	0.51 ± 0.003	0.55 ± 0.03	0.06 ± 0.001
Quercetin 3-*O*-β−D-galactoside	0.53 ± 0.001	0.29 ± 0.01	0.23 ± 0.002
Luteolin 7-*O*-β−D-glucoside	7.37 ± 0.01	nd	nd
Quercetin derivative	0.66 ± 0.003	nd	nd
Quercetin derivative	0.45 ± 0.001	0.52 ± 0.03	nd
Rutin	0.82 ± 0.01	0.42 ± 0.03	nd
***Phenolic acids***			
Gallic acid	3.52 ± 0.32	4.85 ± 0.17	nd
Syringic acid	0.65 ± 0.36	1.24 ± 0.07	nd
Gallic acid derivate	1.08 ± 0.004	3.58 ± 0.24	nd
Gallic acid derivate	0.67 ± 0.05	0.57 ± 0.08	nd
Gallic acid derivate	nd	0.45 ± 0.01	nd
***Chalcone***			
Arbutin	52.33 ± 0.61	7.43 ± 1.14	nd
***Dihydrochalcones***			
Phloridzin	nd	nd	0.51± 0.01

Data are mean ± standard deviation SD (n = 3) and expressed as µg mg_NES_^-1^. nd = not detected.

**Table 5:** Values of IC_50_ of NES. Ascorbic acid was used as positive control for PPO (polyphenoloxidase) and POX (peroxidase) inhibition.

IC_50_	Ascorbic Acid	Strawberry Tree Leaves	Strawberry Tree Branches	Apple Byproduct
**PPO**	0.11 ± 0.02 ^a^ *R*^2^ = 0.998	53.92 ± 2.38 ^b^ *R*^2^ = 0.950	5.97 ± 0.73 ^b^ *R*^2^ = 0.927	127.30 ± 0.89 ^c^ *R*^2^ = 0.977
**POX**	0.52 ± 0.05 ^a^ *R*^2^ = 0.986	0.77 ± 0.10 ^a^ *R*^2^ = 0.999	2.25 ± 0.58 ^b^ *R*^2^ = 0.997	13.86 ± 0.76 ^c^ *R*^2^ = 0.947

Data are mean ± standard deviation SD (n = 3) and expressed as (mg mL^−1^). Different superscript letters differ significantly by LSD test (*p* < 0.05). *R*^2^ represents the coefficient of determination of the non-linear regression calculated for IC_50._
